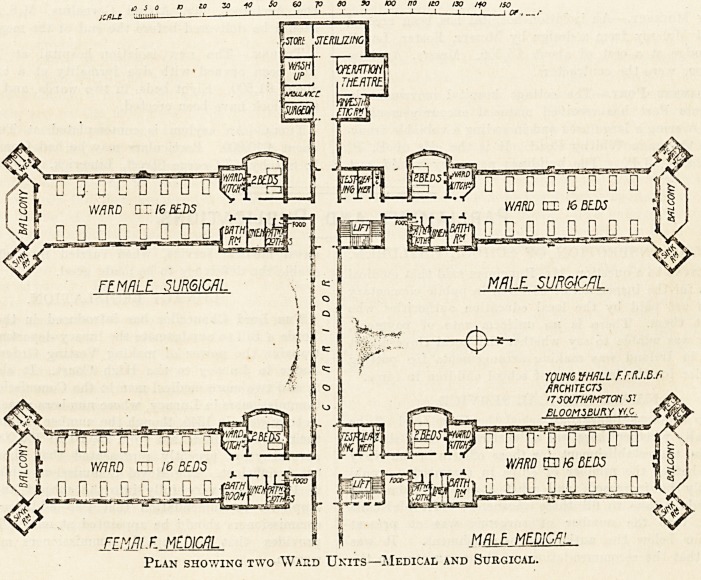# A Complete Ward Unit for a Modern General Hospital

**Published:** 1911-05-27

**Authors:** 


### HOSPITAL ARCHITECTURE AND CONSTRUCTION.

[Communications on this subject should be marked "Architecture" in the left-hand top corner of the envelope.]

### A COMPLETE WARD UNIT FOR A MODERN GENERAL HOSPITAL.

By YOUNG and HALL, FF.R.I.B.A.

This plan shows two complete units, one medical, the other surgical.

### Essentials.

A ward unit to be complete should comprise wards for both sexes, with all the necessary offices, and should be planned with a view to its being under the control of one sister. Opinions as to the maximum number of beds which one sister can properly supervise differ; but if the number is large the sister's duties will more nearly approximate to those of a matron than if they are small enough to allow of her personally supervising the work of her nurses. In fixing the size of the wards at sixteen beds we have had in mind rather the needs of a general hospital in a provincial town of moderate size, and where no medical school exists.

### The Sister's Room.

Another point on which much difference of opinion exists among matrons of great experience is the provision of a sister's room adjoining the wards. The view expressed recently by a matron of a large hospital, who had been trained in one of the largest hospitals in the country, is that where a

Plan showing two Ward Units—Medical and Surgical.

sister has charge of anything like fifty beds or over she should have a small room in which to do the necessary bookkeeping, which forms part of her work, and in which to interview nurses and servants. But it should be an office, and not her sitting-room for use when off duty.

Corridors.

The corridor connecting the two blocks would on the ground floor only be closed in at the sides. This is to afford access for patients from the medical wards to the operation theatre, a necessity which does arise occasionally. On the floors above a covered way open at the sides will suffice.

We have not followed the Glasgow plan in putting the house physician's and house surgeon's rooms within their respective units. It seems to us far better to group the quarters for the residents in the administrative part of the building rather than to scatter them about in the various ward units.

## Figures and Tables

**Figure f1:**